# Chloridobis(1,2,3,4-tetra­hydro-1,4,6,11-tetra­aza­naphthacene-κ*N* ^6^)copper(I)

**DOI:** 10.1107/S1600536812005582

**Published:** 2012-02-17

**Authors:** Xing-Shun Chen, Jing-Jing Zhang, Tai-Ke Duan, Qun Chen, Qian-Feng Zhang

**Affiliations:** a Institute of Molecular Engineering and Applied Chemistry, Anhui University of Technology, Ma’anshan, Anhui 243002, People’s Republic of China; b Department of Applied Chemistry, School of Petrochemical Engineering, Changzhou University, Jiangsu 213164, People’s Republic of China

## Abstract

In the title complex, [CuCl(C_14_H_12_N_4_)_2_], the Cu^I^ atom, lying on a twofold rotation axis, is coordinated by two N atoms of two 1,2,3,4-tetra­hydro-1,4,6,11-tetra­aza­naphthacene ligands and one Cl atom, also lying on the twofold rotation axis, in a distorted trigonal-planar geometry. The complex mol­ecules are connected into a one-dimensional structure along [001] *via* N—H⋯N hydrogen bonds and further into a three-dimensional structure *via* N—H⋯Cl hydrogen bonds. π–π inter­actions between the pyrazine and benzene rings and between the benzene rings [centroid–centroid distances = 3.5635 (15) and 3.9128 (16) Å] are present.

## Related literature

For transition metal complexes with heterocyclic ligands, see: Dai *et al.* (2007 ▶); Grove *et al.* (2000 ▶, 2001 ▶); Näther & Beck (2004 ▶); Xu *et al.* (2011 ▶). For a description of the Cambridge Structural Database, see: Allen (2002 ▶).
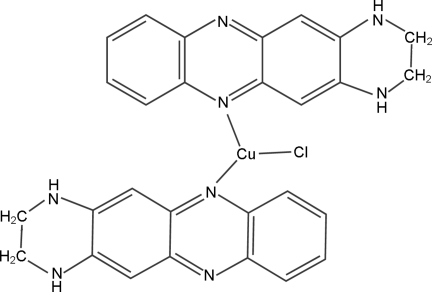


## Experimental

### 

#### Crystal data

[CuCl(C_14_H_12_N_4_)_2_]
*M* *_r_* = 571.54
Monoclinic, 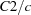

*a* = 16.987 (4) Å
*b* = 11.606 (3) Å
*c* = 14.487 (4) Å
β = 118.492 (3)°
*V* = 2510 (1) Å^3^
*Z* = 4
Mo *K*α radiation
μ = 1.01 mm^−1^
*T* = 296 K
0.29 × 0.24 × 0.06 mm

#### Data collection

Bruker APEX CCD diffractometer
Absorption correction: multi-scan (*SADABS*; Sheldrick, 1996 ▶) *T*_min_ = 0.758, *T*_max_ = 0.942
7571 measured reflections
2839 independent reflections
2476 reflections with *I* > 2σ(*I*)
*R*_int_ = 0.032

#### Refinement

*R*[*F* ^2^ > 2σ(*F* ^2^)] = 0.036
*wR*(*F* ^2^) = 0.105
*S* = 1.03
2839 reflections
173 parameters
H-atom parameters constrained
Δρ_max_ = 0.94 e Å^−3^
Δρ_min_ = −0.34 e Å^−3^

### 

Data collection: *SMART* (Bruker, 2007 ▶); cell refinement: *SAINT* (Bruker, 2007 ▶); data reduction: *SAINT*; program(s) used to solve structure: *SHELXS97* (Sheldrick, 2008 ▶); program(s) used to refine structure: *SHELXL97* (Sheldrick, 2008 ▶); molecular graphics: *SHELXTL* (Sheldrick, 2008 ▶); software used to prepare material for publication: *SHELXTL*.

## Supplementary Material

Crystal structure: contains datablock(s) I, global. DOI: 10.1107/S1600536812005582/hy2514sup1.cif

Structure factors: contains datablock(s) I. DOI: 10.1107/S1600536812005582/hy2514Isup2.hkl

Additional supplementary materials:  crystallographic information; 3D view; checkCIF report

## Figures and Tables

**Table 1:** Hydrogen-bond geometry (Å, °)

*D*—H⋯*A*	*D*—H	H⋯*A*	*D*⋯*A*	*D*—H⋯*A*
N3—H3N⋯N2^i^	0.86	2.22	2.986 (2)	148
N4—H4N⋯Cl1^ii^	0.86	2.76	3.4952 (18)	145

Symmetry codes: (i) 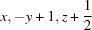; (ii) 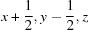.

**Fig. 1.**
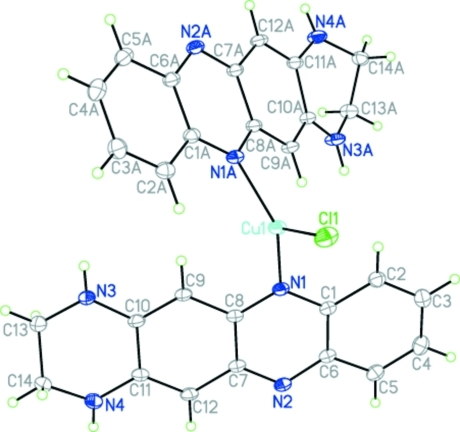
The molecular structure of the title compound, showing displacement ellipsoids at the 50% probability level. [Symmetry code: (A) 1-x, y, 3/2-z.]

**Fig. 2.**
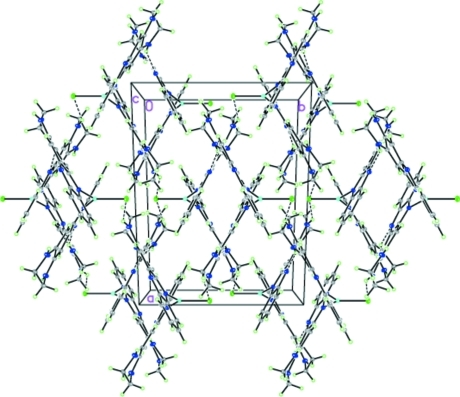
Packing diagram of the title compound viewed along the c axis. N—H···N and N—H···Cl hydrogen bonds are shown as dashed lines.

## supplementary crystallographic information

### Comment

The heterocyclic compounds involving aromatic system with condensed pyrazine, pyridine and piperidine rings have been shown to occur as a rigid bridge in transition metal complexes, which are expected to be good building blocks for creating coordination polymers due to the flexibility of the heterocyclic ligands (Grove *et al.*, 2000, 2001). We have recently been studying the coordination chemistry of polyamines to transition metal halides (Dai *et al.*, 2007, Xu *et al.*, 2011). In the course of this work, we have synthesized the title copper(I) complex with a new 1,2,3,4-tetrahydro-1,4,6,11-tetraazanaphthacene ligand formed from the condensing reaction of phenazine and ethane-1,2-diamine under hydrothermal conditions. Here we report the crystal structure of the mononuclear copper(I) complex.

The molecular structure of the title complex is depicted in Fig. 1. The Cu^I^ atom, lying on a twofold rotation axis, is coordinated by two N atoms of two organic ligands and one Cl atom. The Cu—N bond length of 1.9927 (15) Å and the Cu—Cl bond length of 2.2229 (10) Å are in the range of those found in related structures retrieved from the Cambridge Structural Database (Allen, 2002). The N—Cu—N and N—Cu—Cl angles are 123.94 (9) and 118.03 (4)°. The Cu atom shows a distorted trigonal-planar coordination geometry (Näther & Beck, 2004). In the crystal, the discrete complex molecules are connected by N—H···N and N—H···Cl hydrogen bonds (Table 1) into a three-dimensional structure (Fig. 2). π–π interactions between the pyrazine and benzene rings and between the benzene rings [centroid–centroid distances = 3.5635 (15) and 3.9128 (16) Å] are present.

### Experimental

CuCl (99 mg, 1 mmol), phenazine (360 mg, 2 mmol) and ethane-1,2-diamine (300 mg, 5 mmol) were mixed in water (ca. 3 g) and placed in a 23 ml Teflon-lined stainless steel autoclave and stirred for 20 min. The vessel was sealed and heated to 140°C for 2 d and then cooled to room temperature. Yellow flake crystals were obtained and air dried (yield: 64% based on CuCl). Analysis, calculated for C_28_H_24_ClCuN_8_: C 58.84, H 4.23, N 19.61%; found: C 58.76, H 4.18, N 19.55%.

### Refinement

H atoms were placed in geometrically idealized positions and refined as riding atoms, with C—H = 0.93 (CH) and 0.97 (CH_2_) and N—H = 0.86 Å and with *U*_iso_(H) = 1.2*U*_eq_(C, N).

### Crystal data

**Table:**

[CuCl(C_14_H_12_N_4_)_2_]	*F*(000) = 1176
*M**_r_* = 571.54	*D*_x_ = 1.512 Mg m^−^^3^
Monoclinic, *C*2/*c*	Mo *K*α radiation, λ = 0.71073 Å
Hall symbol: -C 2yc	Cell parameters from 3886 reflections
*a* = 16.987 (4) Å	θ = 2.4–27.4°
*b* = 11.606 (3) Å	µ = 1.01 mm^−^^1^
*c* = 14.487 (4) Å	*T* = 296 K
β = 118.492 (3)°	Flake, yellow
*V* = 2510 (1) Å^3^	0.29 × 0.24 × 0.06 mm
*Z* = 4	

### Data collection

**Table:**

Bruker APEX CCD diffractometer	2839 independent reflections
Radiation source: fine-focus sealed tube	2476 reflections with *I* > 2σ(*I*)
Graphite monochromator	*R*_int_ = 0.032
φ and ω scans	θ_max_ = 27.4°, θ_min_ = 2.3°
Absorption correction: multi-scan (*SADABS*; Sheldrick, 1996)	*h* = −12→22
*T*_min_ = 0.758, *T*_max_ = 0.942	*k* = −15→15
7571 measured reflections	*l* = −18→18

### Refinement

**Table:**

Refinement on *F*^2^	Primary atom site location: structure-invariant direct methods
Least-squares matrix: full	Secondary atom site location: difference Fourier map
*R*[*F*^2^ > 2σ(*F*^2^)] = 0.036	Hydrogen site location: inferred from neighbouring sites
*wR*(*F*^2^) = 0.105	H-atom parameters constrained
*S* = 1.03	*w* = 1/[σ^2^(*F*_o_^2^) + (0.0673*P*)^2^ + 1.176*P*] where *P* = (*F*_o_^2^ + 2*F*_c_^2^)/3
2839 reflections	(Δ/σ)_max_ < 0.001
173 parameters	Δρ_max_ = 0.94 e Å^−^^3^
0 restraints	Δρ_min_ = −0.34 e Å^−^^3^

### Special details

**Table:**

Geometry. All esds (except the esd in the dihedral angle between two l.s. planes) are estimated using the full covariance matrix. The cell esds are taken into account individually in the estimation of esds in distances, angles and torsion angles; correlations between esds in cell parameters are only used when they are defined by crystal symmetry. An approximate (isotropic) treatment of cell esds is used for estimating esds involving l.s. planes.
Refinement. Refinement of F^2^ against ALL reflections. The weighted R-factor wR and goodness of fit S are based on F^2^, conventional R-factors R are based on F, with F set to zero for negative F^2^. The threshold expression of F^2^ > 2sigma(F^2^) is used only for calculating R-factors(gt) etc. and is not relevant to the choice of reflections for refinement. R-factors based on F^2^ are statistically about twice as large as those based on F, and R- factors based on ALL data will be even larger.

### Fractional atomic coordinates and isotropic or equivalent isotropic displacement parameters (Å^2^)

**Table:**

	*x*	*y*	*z*	*U*_iso_*/*U*_eq_	
Cu1	0.5000	0.73465 (3)	0.7500	0.03244 (13)	
Cl1	0.5000	0.92619 (6)	0.7500	0.0465 (2)	
N1	0.54270 (10)	0.65395 (13)	0.66072 (12)	0.0286 (3)	
N2	0.63130 (11)	0.57439 (14)	0.54935 (12)	0.0320 (3)	
N3	0.75994 (11)	0.46038 (17)	0.96600 (13)	0.0410 (4)	
H3N	0.7305	0.4785	0.9984	0.049*	
N4	0.86565 (12)	0.42484 (17)	0.87242 (14)	0.0416 (4)	
H4N	0.8924	0.3918	0.8420	0.050*	
C1	0.50582 (13)	0.66976 (15)	0.55440 (14)	0.0290 (4)	
C2	0.42264 (14)	0.72702 (16)	0.49896 (16)	0.0345 (4)	
H2	0.3934	0.7547	0.5348	0.041*	
C3	0.38508 (16)	0.74169 (17)	0.39227 (17)	0.0391 (5)	
H3	0.3300	0.7785	0.3559	0.047*	
C4	0.42941 (16)	0.7014 (2)	0.33765 (16)	0.0425 (5)	
H4	0.4035	0.7126	0.2655	0.051*	
C5	0.50959 (14)	0.64641 (18)	0.38897 (15)	0.0376 (4)	
H5	0.5379	0.6205	0.3516	0.045*	
C6	0.55040 (13)	0.62826 (16)	0.49911 (14)	0.0305 (4)	
C7	0.66554 (13)	0.55593 (16)	0.65251 (14)	0.0301 (4)	
C8	0.61892 (12)	0.59332 (15)	0.70931 (14)	0.0279 (4)	
C9	0.65396 (13)	0.56262 (16)	0.81577 (14)	0.0317 (4)	
H9	0.6237	0.5853	0.8519	0.038*	
C10	0.73143 (12)	0.50019 (17)	0.86743 (14)	0.0309 (4)	
C11	0.78465 (12)	0.47576 (16)	0.81447 (14)	0.0310 (4)	
C12	0.74970 (13)	0.50098 (17)	0.70967 (15)	0.0340 (4)	
H12	0.7821	0.4815	0.6753	0.041*	
C13	0.83878 (14)	0.38791 (19)	1.01881 (16)	0.0398 (4)	
H13A	0.8230	0.3080	0.9992	0.048*	
H13B	0.8634	0.3945	1.0943	0.048*	
C14	0.90674 (13)	0.42682 (19)	0.98672 (16)	0.0367 (4)	
H14A	0.9268	0.5042	1.0124	0.044*	
H14B	0.9583	0.3760	1.0168	0.044*	

### Atomic displacement parameters (Å^2^)

**Table:**

	*U*^11^	*U*^22^	*U*^33^	*U*^12^	*U*^13^	*U*^23^
Cu1	0.0307 (2)	0.0395 (2)	0.0346 (2)	0.000	0.02160 (15)	0.000
Cl1	0.0614 (5)	0.0364 (4)	0.0555 (4)	0.000	0.0391 (4)	0.000
N1	0.0313 (8)	0.0308 (8)	0.0322 (7)	0.0003 (6)	0.0219 (6)	0.0012 (6)
N2	0.0361 (8)	0.0371 (8)	0.0321 (8)	−0.0009 (6)	0.0238 (7)	−0.0010 (6)
N3	0.0355 (9)	0.0627 (12)	0.0353 (8)	0.0143 (8)	0.0255 (8)	0.0114 (8)
N4	0.0349 (9)	0.0594 (11)	0.0394 (9)	0.0118 (8)	0.0250 (8)	0.0033 (8)
C1	0.0332 (9)	0.0280 (9)	0.0321 (9)	−0.0029 (7)	0.0206 (8)	−0.0004 (6)
C2	0.0368 (10)	0.0321 (10)	0.0396 (10)	−0.0002 (8)	0.0221 (9)	0.0014 (7)
C3	0.0369 (11)	0.0370 (10)	0.0390 (11)	0.0012 (8)	0.0146 (9)	0.0017 (8)
C4	0.0499 (12)	0.0427 (11)	0.0311 (9)	−0.0009 (10)	0.0164 (9)	0.0004 (8)
C5	0.0453 (11)	0.0395 (10)	0.0332 (9)	−0.0018 (9)	0.0230 (9)	−0.0020 (8)
C6	0.0350 (9)	0.0313 (9)	0.0322 (9)	−0.0028 (7)	0.0218 (8)	−0.0015 (7)
C7	0.0332 (9)	0.0337 (9)	0.0325 (9)	−0.0025 (7)	0.0231 (8)	−0.0022 (7)
C8	0.0294 (9)	0.0300 (9)	0.0319 (8)	−0.0019 (7)	0.0208 (7)	−0.0022 (6)
C9	0.0335 (9)	0.0397 (10)	0.0323 (9)	0.0044 (7)	0.0241 (8)	0.0011 (7)
C10	0.0319 (9)	0.0371 (10)	0.0318 (8)	−0.0011 (7)	0.0217 (8)	−0.0011 (7)
C11	0.0305 (9)	0.0343 (9)	0.0366 (9)	0.0003 (7)	0.0229 (8)	−0.0017 (7)
C12	0.0354 (10)	0.0426 (10)	0.0362 (9)	0.0021 (8)	0.0268 (8)	−0.0019 (8)
C13	0.0374 (11)	0.0467 (12)	0.0407 (10)	0.0080 (9)	0.0229 (9)	0.0090 (9)
C14	0.0302 (10)	0.0432 (11)	0.0392 (10)	0.0051 (8)	0.0185 (8)	0.0028 (8)

### Geometric parameters (Å, º)

**Table:**

Cu1—N1	1.9927 (15)	C4—C5	1.360 (3)
Cu1—Cl1	2.2229 (10)	C4—H4	0.9300
N1—C8	1.341 (2)	C5—C6	1.420 (2)
N1—C1	1.370 (2)	C5—H5	0.9300
N2—C7	1.337 (2)	C7—C12	1.417 (3)
N2—C6	1.362 (3)	C7—C8	1.454 (2)
N3—C10	1.352 (2)	C8—C9	1.408 (2)
N3—C13	1.452 (3)	C9—C10	1.370 (3)
N3—H3N	0.8600	C9—H9	0.9300
N4—C11	1.358 (3)	C10—C11	1.466 (2)
N4—C14	1.458 (3)	C11—C12	1.372 (3)
N4—H4N	0.8600	C12—H12	0.9300
C1—C2	1.414 (3)	C13—C14	1.505 (3)
C1—C6	1.423 (2)	C13—H13A	0.9700
C2—C3	1.373 (3)	C13—H13B	0.9700
C2—H2	0.9300	C14—H14A	0.9700
C3—C4	1.407 (3)	C14—H14B	0.9700
C3—H3	0.9300		
			
N1^i^—Cu1—N1	123.94 (9)	N2—C7—C12	120.33 (16)
N1^i^—Cu1—Cl1	118.03 (4)	N2—C7—C8	121.38 (17)
N1—Cu1—Cl1	118.03 (4)	C12—C7—C8	118.28 (16)
C8—N1—C1	118.01 (15)	N1—C8—C9	120.37 (15)
C8—N1—Cu1	117.73 (12)	N1—C8—C7	120.65 (16)
C1—N1—Cu1	123.63 (12)	C9—C8—C7	118.97 (16)
C7—N2—C6	117.44 (15)	C10—C9—C8	121.74 (16)
C10—N3—C13	122.01 (16)	C10—C9—H9	119.1
C10—N3—H3N	119.0	C8—C9—H9	119.1
C13—N3—H3N	119.0	N3—C10—C9	121.64 (16)
C11—N4—C14	119.34 (16)	N3—C10—C11	119.16 (17)
C11—N4—H4N	120.3	C9—C10—C11	119.20 (16)
C14—N4—H4N	120.3	N4—C11—C12	123.55 (16)
N1—C1—C2	119.89 (16)	N4—C11—C10	117.16 (16)
N1—C1—C6	120.43 (17)	C12—C11—C10	119.24 (17)
C2—C1—C6	119.68 (17)	C11—C12—C7	121.74 (16)
C3—C2—C1	119.97 (19)	C11—C12—H12	119.1
C3—C2—H2	120.0	C7—C12—H12	119.1
C1—C2—H2	120.0	N3—C13—C14	108.31 (16)
C2—C3—C4	120.4 (2)	N3—C13—H13A	110.0
C2—C3—H3	119.8	C14—C13—H13A	110.0
C4—C3—H3	119.8	N3—C13—H13B	110.0
C5—C4—C3	120.90 (19)	C14—C13—H13B	110.0
C5—C4—H4	119.5	H13A—C13—H13B	108.4
C3—C4—H4	119.5	N4—C14—C13	108.89 (17)
C4—C5—C6	120.59 (19)	N4—C14—H14A	109.9
C4—C5—H5	119.7	C13—C14—H14A	109.9
C6—C5—H5	119.7	N4—C14—H14B	109.9
N2—C6—C5	119.74 (16)	C13—C14—H14B	109.9
N2—C6—C1	121.78 (16)	H14A—C14—H14B	108.3
C5—C6—C1	118.47 (18)		

Symmetry code: (i) −*x*+1, *y*, −*z*+3/2.

### Hydrogen-bond geometry (Å, º)

**Table:**

*D*—H···*A*	*D*—H	H···*A*	*D*···*A*	*D*—H···*A*
N3—H3*N*···N2^ii^	0.86	2.22	2.986 (2)	148
N4—H4*N*···Cl1^iii^	0.86	2.76	3.4952 (18)	145

Symmetry codes: (ii) *x*, −*y*+1, *z*+1/2; (iii) *x*+1/2, *y*−1/2, *z*.
